# Updating the International Early Warning Score with frailty and comparing to gestalt for prediction of 3-day critical illness and mortality in emergency department patients

**DOI:** 10.1007/s11739-025-04096-x

**Published:** 2025-08-26

**Authors:** Bart G. J. Candel, Lars I. Veldhuis

**Affiliations:** 1 Department of Emergency Medicine, Máxima Medical Centre, Albinusdreef 2, 2333 ZA Veldhoven, The Netherlands; 2 Department of Emergency Medicine, Leiden University Medical Centre, Albinusdreef 2, 2333 ZA Leiden, The Netherlands; 3 Department of Anesthesiology, Erasmus Medical Centre, Rotterdam, The Netherlands; 4 Department of Emergency Medicine, Amsterdam University Medical Centre, Amsterdam, The Netherlands

**Keywords:** Risk stratification, Early warning scores, Mortality, Emergency medicine, Emergency medical services

## Abstract

The International Early Warning Score (IEWS) showed strong prediction of mortality in all ages, but its performance compared to clinical gestalt remains uncertain. Furthermore, it is unknown whether frailty improves prediction besides age. This study aimed to compare IEWS with gestalt, and assess whether updating with frailty improved prediction. This secondary analysis of a prospective study enrolled 774 adult ED patients transported by ambulance to Amsterdam University Medical Centre between March and October 2021. The primary outcome was the performance of IEWS (± frailty) and clinical gestalt of paramedics, nurses, and physicians in predicting critical illness and 28-day mortality. Critical illness included serious adverse events, ICU admission, or mortality within 72 h. Critical illness occurred in 14.1% (n = 109) and 28-day mortality was 7.1% (n = 55). Both gestalt and IEWS had low accuracy predicting mortality with substantial underestimation of risk in all patients and low clinical usefulness. Gestalt performed better than IEWS in terms of discrimination and calibration for critical illness, with AUROC for physicians’ gestalt of 0.83 (0.80–0.86), of nurses’ gestalt of 0.84 (0.81–0.87), and paramedics’ gestalt of 0.78 (0.75–0.81) compared to AUROC for IEWS of 0.64 (0.60–0.69) and IEWS + frailty of 0.64 (0.60–0.69). However, gestalt was only clinically useful for patients in whom an acceptable risk threshold for critical illness was above 5%. In these high-risk patients who arrived by ambulance to an Academic Hospital, clinical gestalt performed better than IEWS plus or minus frailty but was only useful if a risk threshold above 5% was acceptable.

**Supplementary Information:**

The online version contains supplementary material available at 10.1007/s11739-025-04096-x.

## Introduction

Clinical gestalt is the foundation of medical practice and decision-making, and can be considered an intuitive synthesis of experience, patient cues and analytical reasoning [1]. It reflects a complex interplay of intuition and evidence-based analysis that enables rapid assessment and prioritization of patients in dynamic clinical settings. Early Warning Scores (EWSs), on the other hand, are structured prediction tools widely implemented in hospitals to early recognize clinical deterioration of patients and trigger timely interventions, such as intensive care consultation [2–5]. These tools aim to support clinicians by providing objective risks, which may potentially improve outcomes of patients with time-sensitive medical conditions, such as sepsis or trauma [6–9]. In low-risk cases as classified by an EWS, a full clinical evaluation can be postponed, allowing greater attention to be given to high-risk patients who require urgent assessment and treatment. However, for a risk tool to be effective, its predictive performance must at least match, if not exceed, that of clinical gestalt, performed by nurses or physicians.

Recently, a study showed that the Modified Early Warning Score (MEWS) was inferior for predicting 3-day critical illness compared to gestalt, and had a lower specificity for 28-day mortality prediction than clinical gestalt [10]. This could be explained by the fact that MEWS was not developed by applying statistical methods but rather by clinical consensus [2]. In addition, studies which have validated the performance of MEWS or other EWS were found to have methodological weaknesses and inadequate reporting which may have caused serious bias (e.g., inappropriate handling of missing data, poor reporting of event rates, and statistical underpower) [11]. Moreover, predictive performance of MEWS is poor with overestimation of risk in younger ED patients and underestimation of risk in older ED patients [12–14]. For this reason, a recalibrated age and sex-adjusted EWS, the International Early Warning Score (IEWS), was developed. Additionally, the respiratory rate and oxygenation (ROX) index has been suggested for mortality prediction, which performed better than the National Early Warning Score (NEWS) and only includes three vital signs resulting in faster and easier risk stratification [15].

While the IEWS is adjusted for age, it does not account for variations in frailty among older patients. Frailty has been proposed as a better alternative to age, alongside vital signs upon arrival to the ED, and may enhance measures of illness severity for mortality prediction [16, 17]. A recent study demonstrated that including frailty as an additional variable improved predictive performance compared to using NEWS alone [18]. However, the evaluation of frailty represents a significant source of uncertainty: numerous frailty scores exist, but no definitive consensus has been reached on which is optimal. In clinical practice, the identification of frailty often relies on clinician gestalt rather than standardized measurement. Furthermore, it is questionable whether frailty has additional predictive performance if age is already incorporated in a score, such as in the IEWS. Notably, frailty itself could be viewed as a form of ageism due to its perceived chronic nature; and should only be used for mortality prediction if it shows clear performance benefits over the IEWS alone.

The aim of this study was to compare the predictive performance of IEWS, an updated model of IEWS + frailty, ROX index and clinician gestalt for adverse events in patients arriving by ambulance.

## Methods

### Study design and setting

This is a monocentre study conducted at the two ED’s of the Amsterdam University Medical Centre, a tertiary care centre. A retrospective cohort study design was used. Data were extracted from a prior prospective study that included 800 adult ED patients who arrived by ambulance between March 2021 and October 2021 during the hours of 08:00 to 18:00 [10]. This prior study aimed to investigate if critical illness could be recognized by acute healthcare providers. Reporting adheres to the Transparent Reporting of a multivariable model for Individual Prognosis or Diagnosis (TRIPOD) guidelines for prognostic modeling studies [19]. No additional procedures were performed as part of this study. Ethical approval was received by the Medical Ethical Committee (Waiver: W-19_480 # 19.554).

### Patient selection

Participants were selected from the existing dataset based on predefined inclusion and exclusion criteria [10]. For these secondary analyses, patients were excluded if they were lost to follow-up (i.e., due to hospital transfers), were palliative care admissions, or if less than three vital signs were recorded by EMS or at arrival to the ED that were needed to calculate the IEWS as these variables were considered missing not at random and could therefore not be imputed. Patients were followed up for 28 days post-ED admission.

### Data collection

Collected data included the vital signs which were needed to calculate the IEWS, measured prehospital and at ED presentation. Only one set of vital signs was recorded prehospital and at arrival to the ED. Mortality was recorded at day 28 after admission, or patients were called to confirm whether they were alive or deceased. Other variables obtained were disposition (e.g., admission, hospital transfers), serious adverse events (SAE) including ICU admission, sepsis, myocardial infarction, and frailty scores at arrival to the ED and at day 28. The IEWS aggregates seven vital signs: Respiratory Rate (RR) in breaths/min., Systolic Blood pressure (SBP) mmHg, Heart rate (HR) beats/min, peripheral oxygen saturation (SPO2) %, AVPU score, temperature ^0^C, supplemental oxygen (yes or no), and age and sex [20]. The IEWS was calculated for each patient with prehospital vital signs and with vital signs at arrival to the ED. Clinical gestalt for severity of illness, rated on a scale from 0 (not ill) to 3 (severely ill), was recorded from paramedics, nurses, and physicians after the handover from the paramedics to ED staff as described previously [10]. For all patients brought by EMS, a nurse was attending the handover. Physicians were only present at handover in case these patients were not send in by a general practitioner (i.e., prehospital emergency calls and major trauma patients). Several variables were collected for use as predictors in the multiple imputation procedure for missing values: Vital signs after 1 h in the ED, 3 h in the ED, at day 1 after admission, blood analyses performed in ED (Urea, lactate, leukocytes), type of ED bay (normal room, shock room, trauma room, acute brain care, acute cardiac care), and patients’ resuscitation policy.

### Outcome

Primary outcome was the performance of IEWS, IEWS + frailty, ROX index, and clinical gestalt of healthcare providers in predicting the outcome measures three-day critical illness and 28-day mortality and expressed as calibration and discrimination (area under receiver operating characteristics, AUROC) and net benefit. Critical illness was defined as mortality, ICU admission (direct or indirect), sepsis, or myocardial infarction.

### Sample size

The sample size adhered to the rule of thumb for validation of prediction models, requiring a minimum of 100 events and 100 non-events to ensure reliable performance estimates [21]. In the entire dataset, critical illness occurred in 113 patients within 3 days, and 58 patients died within 28 days, providing sufficient events for validating the IEWS.

### Data analysis

Descriptive data were presented as mean (standard deviation (SD)) if normally distributed and median (interquartile range (IQR)) if skewed. Statistical outliners were treated as missing.

Prior to the main analyses, we assessed non-linearity of frailty in univariable logistic regression and explored non-linear terms (restricted cubic splines) for best fit. A missing data analysis was performed to evaluate the feasibility of multiple imputation. Missing data in the cohort were substituted by multiple imputation to reduce information bias [22]. Missing gestalt and vital sign data in the IEWS were imputed using the chained equations procedure, incorporating urea, leukocytes, lactate levels, resuscitation bay, resuscitation policy, outcomes, gestalt, and vital signs in-hospital as predictors. Outcomes were imputed if missing. Twenty imputations were performed, each with five iterations, to ensure convergence and collinearity were addressed.

For each imputation set, we calculated the IEWS and the ROX. For each imputed dataset, IEWS, ROX, and clinical gestalt (paramedics, nurses, physicians) were fitted using logistic regression. Because our outcomes (28-day mortality and critical illness) differ from those used in the original IEWS study (in-hospital mortality), and to allow fair comparison with other scores lacking published coefficients, we retained the original point structures but refitted a single overall coefficient for each model to account for case mix differences. We performed a sensitivity analysis using the original IEWS coefficient and intercept.

Predictive performance of IEWS, IEWS + frailty, ROX (with prehospital vital signs and vital signs at arrival), and clinical gestalt was evaluated using the area under the receiver operating characteristic curve (AUROC) with 95% confidence intervals. Calibration plots were generated, and regression coefficients and intercepts were averaged across imputations.

Decision curves assessed the net benefit of IEWS compared to NEWS and clinical gestalt across risk thresholds [23]. Models were evaluated and compared based on calibration, discrimination, and clinical utility.

All statistical analyses were conducted using R statistical software packages: dplyr (v1.0.7), rms (v6.2), and mice (v4.5). Statistical significance was set at p < 0.05.

### Subgroup analysis

Since many patients were treated in a resuscitation bay or acute care bay, they were already receiving full attention from an experienced physician, making the use of risk stratification tools unlikely to provide additional clinical benefit. Therefore, a subgroup analysis was conducted for patients treated in lower-acuity bays, such as a standard ED room or a cardiac care room. The previously described analyses were repeated for this subgroup. Physician gestalt was not assessed in this analysis as physicians did not listen to the handover for these patients.

## Results

### Patient characteristics

In total, 774 patients were included for analysis. Eleven patients were excluded because less than three vital signs were registered prehospital or at arrival to the ED, five patients were excluded because of a palliative admission, and ten patients were lost to follow-up due to hospital transfers. The majority of the included patients were presented at the normal ED ward (57%), while 11.5% were initially presented at the trauma bay, 13.4% at the acute brain care, and 14.2% at the acute cardiac care department of the ED (see Table 1). Critical illness occurred in 14.1% (n = 109) and 28-day mortality was 7.1% (n = 55).

**Table 1:** Patient characteristics

Total cohort	All (N = 774)
Age, years, median [IQR]	66.0 [52.0–77.0]
Sex, male	432 (55.8%)
*Type of emergency room*	
Normal ED bay	441 (57.0%)
Shockroom	30 (3.9%)
Trauma room	89 (11.5%)
Acute brain care	104 (13.4%)
Acute cardiac care	110 (14.2%)
*Systolic blood pressure (mmHg), median [iQR]*	135 [120–160]
Missing	67 (8.7%)
*Heart rate (bpm), median [IQR]*	80.0 [75.0–100]
Missing	93 (12.0%)
*Respiratory Rate (/min.), median [IQR]*	16.0 [16.0–18.0]
Missing	221 (28.6%)
*Peripheral oxygen saturation (%), median [IQR]*	98.0 [96.0–98.0]
Missing	83 (10.7%)
*Level of consciousness (AVPU)*	
Unresponsive	23 (3.0%)
Pain	20 (2.6%)
Verbal	33 (4.3%)
Alert	633 (81.8%)
Missing	65 (8.4%)
*Temperature (degree Celsius), median [IQR]*	36.7 [36.3–37.2]
Missing	265 (34.2%)
*Supplemental oxygen given*	139 (18.0%)
Clinical Frailty Score (0–10), Median [IQR]	3.00 [2.00–6.00]
Missing	76 (9.8%)
*Gestalt EMS, severity of illness*	
Severely sick	154 (19.9%)
Moderately sick	287 (37.1%)
Mildly sick	171 (22.1%)
Not sick	161 (20.8%)
Missing	1 (0.1%)
*Gestalt nurse, severity of illness*	
Severely sick	111 (14.3%)
Moderately sick	262 (33.9%)
Mildly sick	180 (23.3%)
Not sick	165 (21.3%)
Missing	56 (7.2%)
*Gestalt doctor, severity of illness*	
Severely sick	43 (5.6%)
Moderately sick	62 (8.0%)
Mildly sick	37 (4.8%)
Not sick	55 (7.1%)
Missing	577 (74.5%)
Urea (mmol/L), median [IQR]	6.70 [4.90–10.1]
Missing	335 (43.3%)
Leukocytes (*10^9), Median [IQR]	8.40 [6.60–11.6]
Missing	164 (21.2%)
*Lactate (mmol/L), Median [IQR]*	2.10 [1.40–3.48]
Missing	652 (84.2%)
*Resuscitation policy*	
Full	278 (35.9%)
No CPR	29 (3.7%)
No CPR, no ventilation	12 (1.6%)
No CPR, no ventilation, no ICU	57 (7.4%)
Missing	398 (51.4%)
Hospital admission	378 (48.8%)
3-day Critical Illness, N (%)	109 (14.1%)
28-day mortality, N(%)	55 (7.1%)
Missing	29 (3.7%)

The vital signs presented in this table are measured prehospital by the paramedics. *SD* standard deviation, *IQR* interquartile range, *EMS* emergency medical services, *CPR* cardiopulmonary resuscitation

### Main results

Frailty was incorporated as a linear function based on its fit and association with critical illness (Figure E1).

**Fig. 1:**
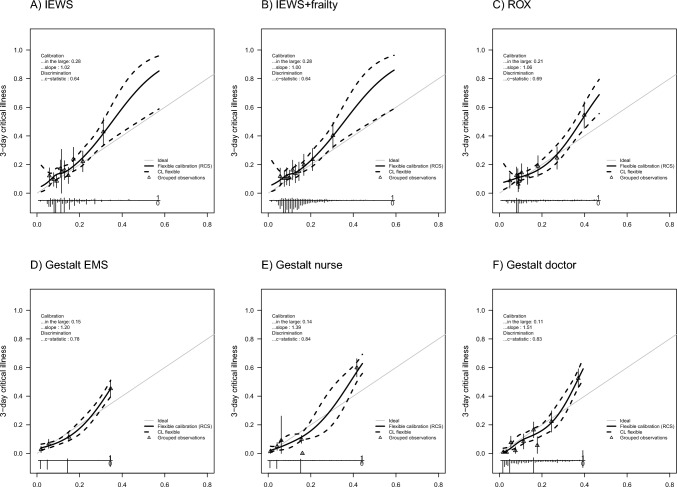
Flexible calibration plots for predicting 3-day critical illness in the total cohort (N = 774) for the International Early Warning Score (IEWS), IEWS + frailty, the Respiratory and oxygenation index (ROX), gestalt of Emergency Medical Services, nurses and physicians

**Fig. 2:**
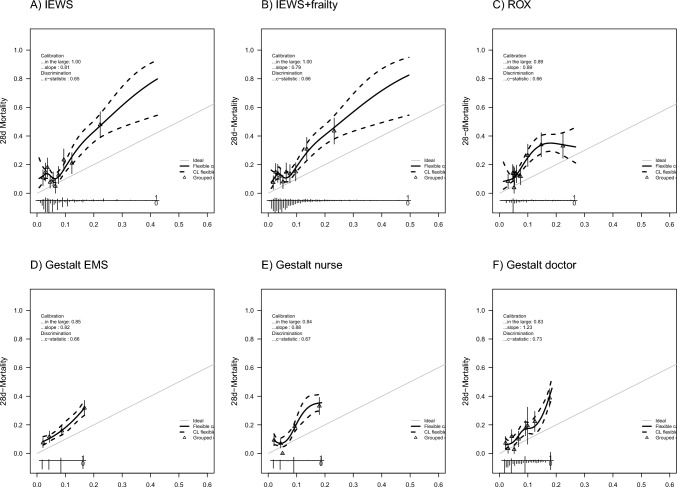
Flexible calibration plots for predicting 28-day mortality in the total cohort (N = 774) for the International Early Warning Score (IEWS), IEWS + frailty, the Respiratory and oxygenation index (ROX), gestalt of Emergency Medical Services, nurses and physicians

For 3-day critical illness, all models exhibited good calibration within the predicted risk range of 0–20%, see Fig. 1. However, at higher risk levels, IEWS and IEWS + frailty significantly underestimated risk. Calibration plots for all models for the 28-day mortality outcome demonstrate poor calibration with substantial underestimation of risk, see Fig. 2.

Gestalt assessments by EMS, nurses, and doctors outperformed all risk models in discriminating 3-day critical illness. Discrimination as expressed as AUROC was 0.78 (0.75–0.81) for EMS, 0.84 (0.81–0.87) for nurse, and 0.83 (0.80–0.86) for doctor gestalt, compared to 0.64 (0.60–0.69) for both IEWS and IEWS + frailty, and 0.69 (0.65–0.74) for ROX. Using vital signs at arrival to the ED yielded similar results as using prehospital vital signs. Full discrimination and calibration metrics for all models are provided in Table 2. Adding frailty as an additional variable to IEWS did not enhance calibration or discrimination.

**Table 2:** Discrimination and Calibration measures for all models

	Calibration		Discrimination	
	Intercept	Slope	AUROC	95% CI
*3-day critical illness, total cohort*				
IEWS	0.28	1.02	0.64	0.60–0.69
IEWS + frailty	0.28	1.00	0.64	0.60–0.69
ROX	0.21	1.06	0.69	0.65–0.74
Gestalt EMS	0.15	1.20	0.78	0.75–0.81
Gestalt Nurse	0.14	1.39	0.84	0.81–0.87
Gestalt Doctor	0.11	1.51	0.83	0.80–0.86
*28-day mortality, total Cohort*				
IEWS	1.00	0.81	0.65	0.60–0.69
IEWS + frailty	1.00	0.79	0.66	0.62–0.71
ROX	0.89	0.89	0.66	0.62–0.70
Gestalt EMS	0.85	0.82	0.66	0.62–0.70
Gestalt Nurse	0.84	0.88	0.67	0.64–0.71
Gestalt Doctor	0.83	1.23	0.73	0.69–0.77
*3-day critical illness, subgroup*				
IEWS	0.02	1.04	0.70	0.66–0.76
IEWS + frailty	0.02	1.03	0.71	0.65–0.77
ROX	− 0.04	1.13	0.61	0.54–0.69
Gestalt EMS	− 0.06	1.19	0.68	0.62–0.73
Gestalt Nurse	− 0.06	1.39	0.74	0.69–0.80
*28-day mortality, subgroup*				
IEWS	0.92	0.76	0.66	0.62–0.70
IEWS + frailty	0.95	0.59	0.68	0.62–0.74
ROX	0.79	0.88	0.62	0.55–0.68
Gestalt EMS	0.77	0.93	0.65	0.60–0.70
Gestalt Nurse	0.76	0.98	0.63	0.58–0.68

*IEWS* International Early Warning Score; *ROX* respiratory and oxygenation index, *EMS* emergency medical services, *AUROC* area under the receiving operating curve

Total cohort contained 774 patients. The subgroup includes 551. patients treated in low acuity bays

Overall, discrimination for all models ranged from poor to moderate, with AUROC values between 0.65 (0.60–0.69) for IEWS and 0.73 (0.69–0.77) for doctor gestalt. Excluding patients treated in resuscitation bays improved IEWS calibration and discrimination for predicting 3-day critical illness but reduced the predictive performance of gestalt (see Figure E2 for calibration plots and Table 2). Both calibration and discrimination for 28-day mortality were poor across all models and gestalt in the subgroup (see Figure E3 for calibration plots).

**Fig. 3:**
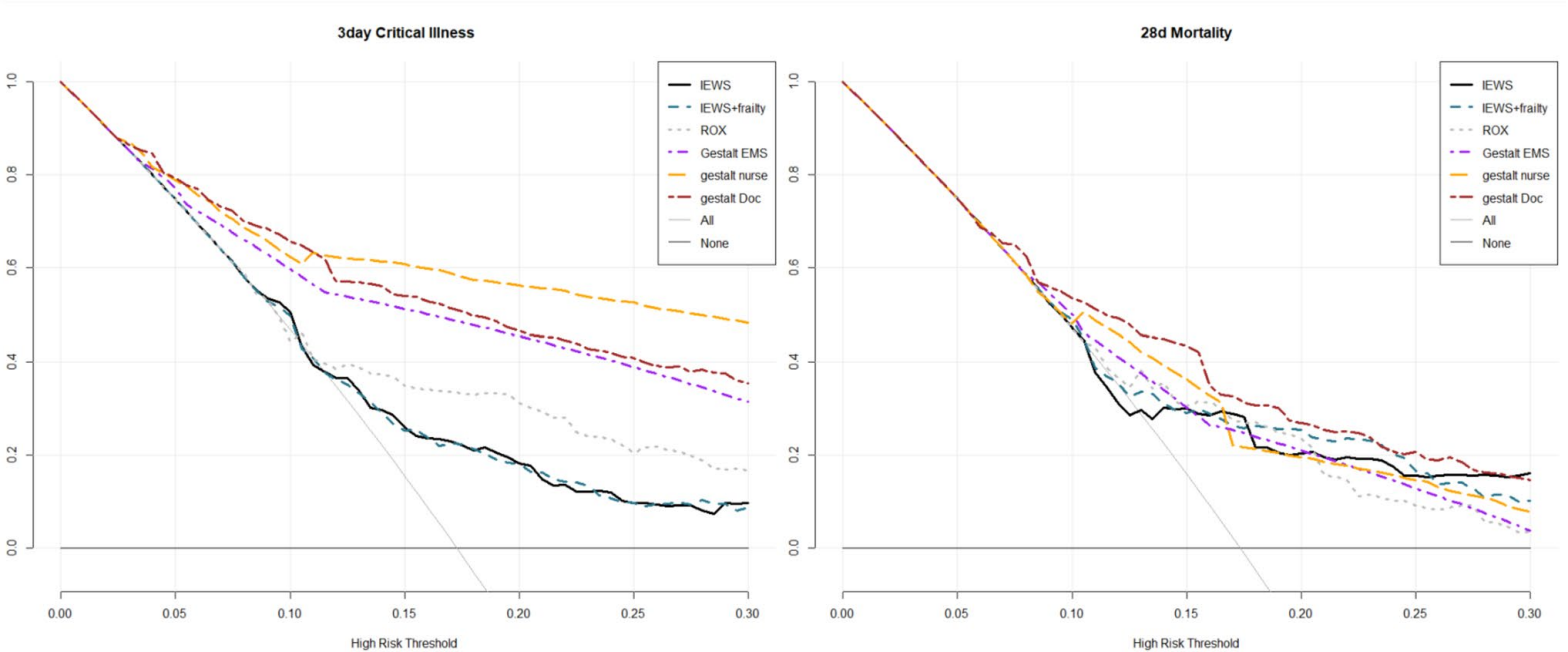
Decision curve analyses for prediction of 3-day critical illness (left panel) and 28-day mortality (right panel) in the risk range of 0 to 30% in the total cohort (N = 774) for the International Early Warning Score (IEWS), IEWS + frailty, the Respiratory and oxygenation index (ROX), gestalt of Emergency Medical Services, nurses and physicians

Decision curve analysis showed that EMS, nurse, and doctor gestalt provided superior clinical utility for predicting 3-day critical illness, particularly within the 5–20% risk threshold, (see Fig. 3 for net benefit analyses). If a risk threshold below 5% is deemed acceptable, all patients should be classified as high risk, rendering risk tools and gestalt assessments unnecessary. For 28-day mortality, gestalt showed a slight advantage over risk scores but only outperformed them when a 10% risk threshold was considered acceptable. Within the relevant risk range for 28-day mortality (3–10%), neither gestalt, IEWS, nor ROX provided additional clinical value in this population.

### Subgroup and sensitivity analyses

Among patients treated in standard ED rooms or cardiac care bays, IEWS performed comparably to gestalt of nurses and EMS for predicting 3-day critical illness (5–12% risk range, see Figure E4). For higher risk thresholds (12–20%), nurse gestalt provided the highest predictive accuracy. Net benefit for mortality prediction did not change in the subgroup compared to the main analyses for the risk models and gestalt (Figure E5). Using the coefficient and intercept of the original IEWS model did not affect discrimination, calibration or net benefit for both outcomes (Figure E6).

## Discussion

Among high-risk ED patients transported by ambulance, IEWS and ROX demonstrated poor predictive value for both 3-day critical illness and 28-day mortality. Clinical gestalt demonstrated good predictive performance and net benefit for critical illness but not for mortality. Adding frailty to IEWS did not improve prediction. In a subgroup of less severely ill patients, risk scores performed similarly to gestalt for critical illness within the relevant risk range, but neither performed well for mortality. These findings highlight the limitations of early warning scores and the minimal impact of frailty in predicting critical illness and mortality in this high-risk ED population.

These findings contrast with a previous development and validation study where IEWS demonstrated strong predictive performance for mortality in a large Dutch cohort of 95,553 patients, as well as in a Danish validation cohort of 14,809 patients [20]. A split-sample analysis within the larger cohort showed that IEWS performed similarly across tertiary care centres and peripheral hospitals. Additionally, IEWS was clinically useful, providing a high net benefit at a predicted risk threshold of just 1%, compared to 10% in our study. Several factors may explain these discrepancies. One possible explanation is differences in case mix, as only about one-third of patients in the previous study arrived by ambulance. In contrast, our cohort, particularly in a tertiary care setting, likely includes a higher proportion of critically ill patients with conditions such as trauma, stroke, or pre-cardiac arrest states. This is reflected in the mortality rate of 7.1% in our study, nearly three times higher than the 2.4% observed in the development cohort of IEWS [20]. In this case mix, age, an important component of IEWS, may not be as important for prediction, since even young patients arriving by ambulance are at high risk for adverse events. As a result, IEWS may not effectively discriminate between patients with low and high risks. Another explanation is that we used 28-day mortality instead of in-hospital mortality. The median length of stay in the development paper was four days. Using a different outcome with a longer time-horizon likely affected predictive performance.

While IEWS did not perform well for mortality prediction in our study, neither did gestalt. However, gestalt outperformed risk scores for predicting critical illness, likely due to its ability to capture early clinical impressions that influence disposition decisions. Since direct ICU admission is a major component of 3-day critical illness, clinicians' gestalt assessments at ED arrival may largely reflect disposition decisions. Supporting this, a previous study found that a predictive model using variables collected two hours after ED arrival did not perform better than one using variables available at arrival for predicting hospital admission [24]. Thus, gestalt's effectiveness may stem more from its ability to anticipate ICU admission rather than directly assessing illness severity. The subgroup analysis showed that in a case mix of less acutely ill patients, gestalt and risk tools performed similarly. Therefore, it is likely that risk tools remain beneficial in lower-risk populations, such as most ED patients. Furthermore, IEWS was not developed to predict critical illness, in which age likely does not play an important role. Our finding that gestalt performed better for predicting critical illness aligns with a previous study comparing gestalt with MEWS in the same cohort as in this study [10]. In the prior study, clinical usefulness was not assessed by decision curve analysis. For a 5% risk threshold, gestalt provided superior net benefit over risk scores, reinforcing its clinical utility. Nonetheless, it is likely that a lower-risk threshold should be applied for many patients, such as young individuals without comorbidities. In such cases, neither gestalt nor risk tools performed well, and all these patients arriving by ambulance to a tertiary care centre should be considered high risk.

Since the ROX index includes the most essential physiological variables to detect deterioration, it may be a helpful risk tool in the ED [15]. Although ROX showed slightly better discrimination and calibration than IEWS for critical illness prediction, there was no advantage in clinical benefit compared to gestalt.

Frailty has been proposed as an additional variable for mortality prediction [16, 25–27]. While previous studies have shown that frailty provides additional predictive value alongside vital signs [16–18], it remained unclear whether frailty adds value besides age as a variable in a risk tool. This study, however, did not demonstrate that frailty improved predictions for critical illness or mortality. Nonetheless, it may still prove beneficial in a different patient case mix.

Our study suggests that mortality prediction in this high-risk patient subset is not improved using gestalt, IEWS, or ROX. All patients arriving by ambulance to this tertiary care centre should be considered high risk. This study underscores the need for further validation to assess the generalizability of IEWS across different settings before widespread use [28, 29]. Additionally, the implementation of risk scores should be accompanied by evaluations of their impact on patient outcomes, such as length of stay, disposition, critical illness, or mortality. EWSs are designed for prognostication and can be used as early as in the ED and add to the clinical evaluation of a patient’s disease severity [30]. While clinical evaluation may vary among physicians depending on years of experience, [31] the IEWS provides a numerical mortality risk (a percentage) that hypothetically may help with clinical decision-making. While gestalt performed better in predicting critical illness, it remains unclear which risk thresholds are acceptable for ED patients and whether these thresholds vary among individuals. For instance, an older patient may have an acceptable risk threshold of 10%, while for a younger patient, it may be as low as 1%. These thresholds likely depend on what is deemed acceptable by both healthcare professionals and patients. Future studies should explore which risk thresholds should prompt more aggressive treatment or decisions for higher care disposition, and whether this varies based on factors such as age, comorbidities, or frailty.

The present study has several strengths. We adhered to the TRIPOD guidelines and followed steps recommended for the validation of prediction models [11, 19, 32]. Several limitations merit emphasis. The key limitation of this study is a highly selected population of one tertiary care centre of patients arriving by ambulance during day hours. This setting excludes many standard ED presentations (e.g., hip fractures, mild infections) as such patients are typically directed to nearby peripheral hospitals. As a result, the case mix primarily includes high-acuity patients such as those with major trauma, shock, intoxications, or complex conditions known to subspecialists. While this limits generalizability, this study highlights the importance of validating risk scores and clinical gestalt in the specific populations where they are applied, before implementation. This reflects a broader, common limitation of many existing risk scores. Second, although we met the recommended 100 events of critical illness for validation, the lower mortality count (n = 55) may have influenced results. Third, a risk of selection bias may be present as we excluded patients who arrived during nighttime. Patients arriving by night may have different disease presentations (i.e., intoxications) and gestalt or risk tools may perform differently. However, for this study, it was not feasible to include patients during evening or nighttime as this would add administration load to the clinical staff. Lastly, some variables were missing, but mostly because they were not recorded by the clinical staff. As recommended, we used multiple imputation to prevent information bias so we could include as many patients as possible if this was deemed possible [11].

In summary, in this high-risk population of patients arriving by ambulance to the ED of a tertiary academic centre, both IEWS and ROX demonstrated poor predictive performance and limited clinical value for predicting 3-day critical illness and 28-day mortality. Clinical gestalt showed good predictive performance for critical illness but not for mortality. However, for patients in whom risk thresholds lower than 5% are acceptable, gestalt did not perform better than considering everyone as high risk. Adding frailty to IEWS did not improve prediction, and in a subgroup of less severely ill patients, risk scores and gestalt performed similarly for critical illness but not for mortality. Although our results may not be generalizable, our findings highlight the limitations of early warning scores and the negligible role of frailty in predicting critical outcomes in an ED population.

## Supplementary Information

Below is the link to the electronic supplementary material.

Supplementary file1 (DOCX 585 KB)

## Data Availability

Data are available upon reasonable request.
