# Supplementary material for: Updating the International Early Warning Score with frailty and comparing to gestalt for prediction of 3-day critical illness and mortality in emergency department patients
Source: Intern Emerg Med. 2025 Aug 26;21(5):1827–35. doi: 10.1007/s11739-025-04096-x (PMC13452842; doi:10.1007/s11739-025-04096-x)
Supplement: Supplementary file 1 [file 11739_2025_4096_MOESM1_ESM.docx]

Authors: Bart G.J. Candel, MD, PhD, Lars I. Veldhuis, MD, PhD

**Online-only supplemental**

Figure E1 The association between frailty and 3-day critical illness	P. 2

Figure E2 Flexible calibration plots for subgroup 28-day mortality	P. 3

Figure E3 Flexible calibration plots for subgroup 28-day mortality	P. 4

Figure E4 Net benefit analysis in subgroup for 3-day critical illness	P. 5

Figure E5 Net benefit analysis in subgroup (N=551) for 28-day mortality	P. 6

Figure E6 comparing recalibrated IEWS with original IEWS model	P. 7

**Figure E1 The association between frailty and 3-day critical illness**

A) *Scatterplot of clinical frailty scores (0 to 10) and mean 3-day critical illness.*

***B) Set 1 linear verband (AUC 0.68, R2 0.07, brier 0.05)***

*Set2 linear boven de >5 frailty (AUC 0.66, R2 0.07, brier 0.05)*

*Set 3 restricted cubic spine (AUC 0.67, R2 0.07, brier 0.05)*

*Set 4 dichotoom <=5 versus >5 (AUC 0.065, R2 0.06, brier 0.05)*

*The linear association was chosen as best fit.*

**Figure E2 Flexible calibration plots for subgroup 3-day critical illness**

**Figure E3 Flexible calibration plots for subgroup 28-day mortality**

**Figure E4 Net benefit analysis in subgroup (N=551) for 3-day critical illness**

**Figure E5 Net benefit analysis in subgroup (N=551) for 28-day mortality**

**Figure E6 comparing recalibrated IEWS with original IEWS model**

**3-day critical illness**

The IEWS in the study was recalibrated. Here we compare this recalibrated IEWS with the original IEWS using the coefficient and intercept of the original model.

IEWS recalibrated: Odds critical illness = e^((0.182*IEWS) – 3.88)^

IEWS original: Odds critical illness = e^((0.384*IEWS) – 6.815)^ AUC 0.64 (0.59-0.68)

IEWS+frailty: Odds critical illness = e^((0.186*IEWS )– (0.084*frailty) – 4.193)^

**28day mortality**

IEWS recalibrated: Odds mortality = e^((0.230*IEWS) – 5.287)^

IEWS original: Odds mortality = e^((0.384*IEWS) – 6.815)^	AUC 0.64 (0.60-0.69)

IEWS+frailty: Odds mortality = e^((0.204*IEWS )– (0.476*frailty) – 3.582)^
